# Sensor-Enabled Nested Networked Control for Speed Synchronization and Swing Damping in Air–Ground Collaborative Distribution

**DOI:** 10.3390/s26010092

**Published:** 2025-12-23

**Authors:** Jingwen Huang, Haina Wang

**Affiliations:** School of Logistics, Beijing Wuzi University, Beijing 101126, China; 24312086100015@bwu.edu.cn

**Keywords:** nested networked control systems, sensor noise, speed synchronization, swing angle stabilization, air ground collaborative distribution

## Abstract

With the rapid development of the low-altitude economy, UAV logistics delivery systems have garnered widespread attention due to their flexibility and efficiency. The cooperative delivery mode involving a UAV with a suspended payload and a ground vehicle represents a typical networked distribution scenario, whose performance is constrained by the tight coupling of sensing, communication, and control. In practical applications, sensor measurement noise and sudden disturbances propagate through the closed-loop system, severely degrading velocity synchronization and swing angle stability. To address this challenge, this paper focuses on a quadrotor UAV slung-load system and proposes a three-layer nested networked closed-loop control architecture for simultaneous velocity tracking of a moving ground target and swing angle stabilization. First, by establishing the system’s dynamic model, the mapping relationship between cable tension and the payload swing angle (based on sensor feedback) is revealed. Then, by setting the payload velocity as the outermost control objective and constructing a coupled error to drive a virtual swing angle actuator, the direct impact of noise in the raw sensor data is effectively mitigated. Subsequently, the desired acceleration of the UAV is derived through inverse computation, achieving synchronous optimization of velocity tracking and swing angle suppression. Theoretical analysis using Lyapunov methods demonstrates the stability of the closed-loop system in the presence of bounded delays. Simulation results show that the proposed method effectively suppresses payload swing, controls velocity synchronization error, and exhibits strong robustness against sensor noise and sudden disturbance. This study provides a control solution that improves the precision and robustness of sensor-enabled networked control systems in complex dynamic scenarios

## 1. Introduction

In recent years, the logistics industry has experienced explosive growth. Traditional delivery models increasingly reveal their efficiency bottlenecks and lack of flexibility when confronting scenarios such as urban traffic congestion, remote area coverage, and emergency material delivery. The rapid rise of the low-altitude economy has injected revolutionary momentum into the logistics sector. Compared to traditional methods, unmanned aerial vehicles (UAVs), leveraging their three-dimensional mobility and cost advantages, are gradually becoming a key pillar of the new logistics system. Particularly within the air-ground collaborative distribution framework, UAVs and ground vehicles form a typical networked control system connected via wireless communication networks, offering an innovative paradigm to overcome the “last-mile” challenge problem [1,2].

In the air-ground collaborative distribution scenario, UAVs are responsible for rapid point-to-point aerial transportation, while unmanned ground vehicles handle high-capacity transfer and final-stage distribution. Under emergency conditions, seamless cargo transfer between them can be achieved without stopping. However, this networked control system faces severe challenges in practical operation: Firstly, the slung-load operation introduces strongly nonlinear, underactuated, and multi-body coupling characteristics, exposing the UAV control to external disturbances and model uncertainties. Secondly, the cargo swing is deeply coupled with the UAV’s pose, making it difficult for traditional single-objective controllers to simultaneously achieve tracking accuracy and stability. More critically, in dynamic collaboration, the UAV state and payload swing angle information—dependent on sensor feedback—are transmitted via a noisy air-ground communication link and are subject to sudden external disturbances. These practical factors, namely sensor noise and sudden disturbances, make it challenging for the controller to accurately and in real time match the ground platform’s velocity while consistently suppressing payload swing throughout the process.The effectiveness and robustness of the control strategy become the core factors determining system performance.

Extensive research has been conducted on quadrotor cable-suspended load systems. For external disturbances in complex environments, some works adopt reinforcement learning strategies based on PPO-Clip with reward functions that combine position tracking and anti-swing performance [3], to improve system robustness and stability under fault scenarios. To mitigate the impact of sensor noise, a nonlinear coupling control strategy is designed to suppress the payload swing in cable-driven UAVs with time-varying cable lengths, thereby blocking the propagation of noise through the system [4]. Energy-integral combined neural networks with online estimation [5] have significantly enhanced system robustness against payload mass variations. In addition, active disturbance rejection control (ADRC) has been used [6] to assist in supplementing total disturbance compensation through expanded state observation.

In terms of tracking accuracy and stability coordination, research has covered both open-loop and closed-loop strategies. The open-loop strategy primarily focuses on trajectory planning. For instance, S-curve trajectory planning for secondary swing suppression effectively integrates both precision tracking and swing suppression [7]; In another approach, Reference [8] constructs auxiliary signal parameters under variable payload scenarios, which enhances the UAV-load coupling and achieves collaborative position-swing optimization. However, open-loop methods exhibit inherent limitations in compensating for real-time disturbances, which motivates the adoption of closed-loop control as the mainstream approach. Based on LQR, the swing suppression controller [9] performs well in linearized models, but nonlinear adaptability is limited. As an alternative, Nonlinear Model Predictive Control (NMPC) is employed in [10] to handle complex payload oscillations, with simulations confirming its robustness advantage over Linear Quadratic Regulator (LQR) methods. In parallel, energy-based nonlinear anti-swing controllers for multi-body systems represent another active research direction [11], demonstrating capabilities for precise position tracking and rapid swing suppression across diverse flight regimes. Furthermore, when integrated with event-driven control and adaptive evaluation networks [12,13], this line of research further reduces the computational burden on the payload, thereby enabling online trajectory optimization for both position and swing dynamics.

For velocity control, Backstepping and Sliding Mode Control (SMC) are widely adopted to achieve stable velocity tracking in vertical-lift UAVs through the construction of layered virtual controllers [14]. Inverse dynamics provides an alternative approach by directly decoupling the system dynamics to facilitate velocity tracking objectives [15]. In industrial settings, reference model integral SMC [16] has been shown to maintain robust velocity tracking even under extreme operating conditions. In the context of multi-UAV coordination and efficiency optimization, the velocity control problem for large-scale UAV systems has been formulated as a micro-differential game in [17], where the G-prox PDHG algorithm is utilized to simultaneously optimize energy consumption, communication performance, and obstacle avoidance. Meanwhile, Nonlinear Model Predictive Control (NMPC) has been applied to achieve full-envelope velocity tracking for tilting-rotor UAVs, with experimental flight tests validating its superior capability in complex maneuvers [18]. Specialized mission scenarios further impose stringent requirements on velocity control. For example, ref. [19] investigates the serial coupling among attitude, swing, and velocity in cable-suspended load systems, achieving effective suppression of multi-axis inclination. Separately, Ref. [20] introduces a hierarchical sliding mode observer (HSMO) for flying-wing UAVs to estimate composite disturbances, demonstrating that integrated dynamic compensation significantly improves command tracking accuracy.

Although existing research has achieved notable progress in swing suppression and velocity control, most studies focus on the problem of position tracking or swing suppression. There has been little systematic effort to address the challenge of synchronizing velocity tracking and payload swing suppression under the aerial-ground collaborative scenario. More critically, in such a closed-loop network composed of sensors, communication links, and controllers, noise and disturbances do not originate solely from the external environment. The control network itself constitutes a complex signal transmission channel, inevitably introducing computational noise, quantization errors, and amplifying and propagating sensor noise within the loop. This implies that while traditional control strategies address external disturbances, they often overlook the degrading effect of their own signal pathways on control quality. In summary, the precise control of air-ground collaborative slung-load systems faces three coupled challenges: (1) the system’s inherent strong coupling and underactuation; (2) the internal noise and disturbance transmission problem introduced by the networked loop formed by the sensors, communication, and controllers themselves; and (3) the dual-objective conflict between velocity tracking and swing suppression. Existing research mostly focuses on the first two points, without systematically treating the control network itself as a dynamic object that needs to be ’shaped’.

Driven by the aforementioned challenges, this paper proposes a three-layer nested networked closed-loop control architecture. The core idea of this architecture is to actively manage and reshape the internal signal pathways of the control network, thereby suppressing internal noise transmission while robustly countering external disturbances. Specifically: the outermost layer takes the load velocity as the primary control variable to ensure tight synchronization with the ground vehicle; the middle layer regards the swing angle as a virtual actuator, actively shaping the cable tension command by constructing a coupled error to attenuate noise propagation; the innermost layer converts the desired angular acceleration into the UAV body acceleration via dynamic inversion computation, which is then executed by the low-level attitude/thrust loop. This nested structure systematically decouples the ’fast’ UAV dynamics from the ’slow’ load motion, provides explicit interfaces for filtered sensor feedback, and allows each layer to be robustly tuned against bounded disturbances—features that are difficult to achieve with conventional single-loop or cascade designs.

## 2. System Analysis and Modeling

### System Description

**Figure 1:**
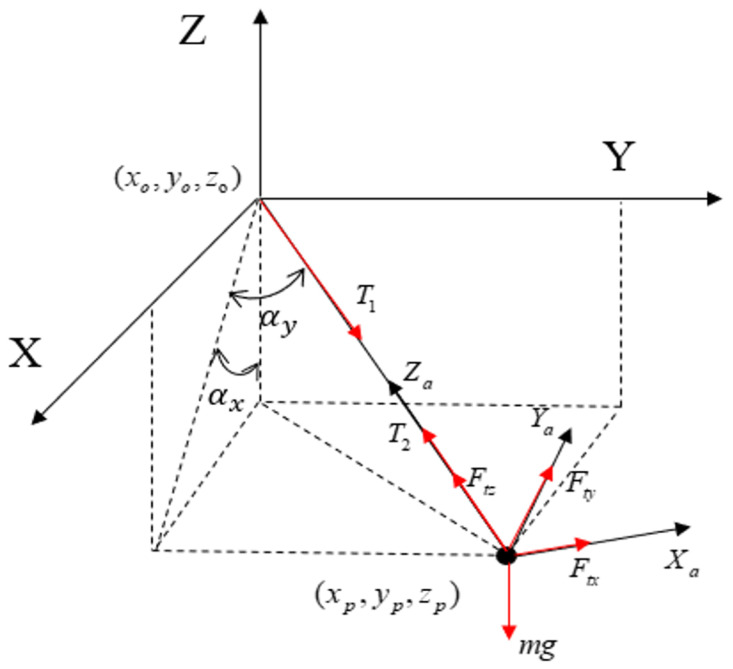
The Inertial Coordinate System.

As illustrated in Figure 1, a quadrotor UAV transports a payload of mass $m_{p}$ connected by a rigid cable of length *L*. The inertial coordinate system O-XYZ is established, with the following key states measured through integrated sensor systems: the UAV position $\xi ={\left[x,y,z\right]}^{⊤}$ via GPS/IMU sensors, the payload position $\delta ={\left[x_{p},y_{p},z_{p}\right]}^{⊤}$ from vision-based sensing, the payload velocity $v_{p}={\left[{\dot{x}}_{p},{\dot{y}}_{p},{\dot{z}}_{p}\right]}^{⊤}$ through sensor fusion, and the payload swing angles $\sigma ={\left[\alpha_{x},\alpha_{y}\right]}^{⊤}$ via inertial measurement units. The following assumptions are made for system modeling:

- The payload is treated as a point mass with negligible moment of inertia.
- The cable linking the UAV and payload is considered rigid.
- The thrust generated by the UAV is directly controllable.
- The swing angles of the payload are confined to σ∈(−π/2,π/2) to prevent singular configurations.

**Assumption Justification.**

- *Point-mass payload.* Treating the payload as a point mass with negligible moment of inertia retains only the kinematic variables (3-D position and velocity) and completely removes the rotational dynamic states. Consequently, the system order is reduced from 12 (6 rigid-body + 6 rotational) to 6 (3-D position + 3-D velocity), halving the model dimension and greatly facilitating real-time computation and subsequent stability proofs.
- *Rigid cable.* By neglecting cable elasticity, the UAV–payload system can be described by a simple pendulum model of constant length *L*. The tension *T* then becomes an algebraic function of the swing angle α, which is essential for the analytic inversion. If axial elasticity were included, *T* would become an additional differential state, introducing extra zero-dynamics and significantly increasing controller complexity.
- *Directly controllable thrust.* Stripping out the motor/ESC/propeller dynamics lets the controller focus on the rigid-body motion only. The motor time-constant (≈15ms) is one order of magnitude faster than the pendulum mode, so the assumption is standard in sling-load literature and reduces the design to a single rigid-body layer.
- *Swing angle range σ∈(−π/2,π/2).* Limiting |σ|<π/2 prevents the horizontal component of tension from approaching zero, avoids controllability singularities, guarantees positive tension, and keeps all trigonometric inversions well-defined.

The payload exhibits motion in three dimensions. The Euler angles $\zeta_{p}$ of the payload rotation about the *X*, *Y*, and *Z* axes are defined as:

(1) $$\zeta_{p}={\left[\phi_{p},\;\theta_{p},\;\psi_{p}\right]}^{⊤}$$

where $\phi_{p}$, $\theta_{p}$, and $\psi_{p}$ represent the payload’s roll, pitch, and yaw angles, respectively.

A correspondence exists between $\zeta_{p}$ and the previously defined payload swing angles $\alpha_{x}$ and $\alpha_{y}$, given by:

(2) $$\phi_{p}=\alpha_{y},\;\theta_{p}=-\alpha_{x},\;\psi_{p}=\psi_{p}$$

Here, the yaw angle $\psi_{p}$ denotes the rotation angle about the cable direction.

The interaction forces transmitted through the tether are denoted as $T_{1}$ (acting on the UAV) and $T_{2}$ (acting on the payload). According to Newton’s third law,

(3) $$T_{1}=-T_{2}$$

Based on Newton’s second law, the translational equations of motion for the UAV and payload in the inertial frame are given by:

(4) $$\begin{array}{c} Ma=F-T_{1}-k_{\alpha }v+G_{1} \end{array}$$

(5) $$\begin{array}{c} m_{p}a_{p}=T_{2}+G_{2} \end{array}$$

where $F={\left[F_{x},F_{y},F_{z}\right]}^{⊤}$ denotes the total thrust vector of the UAV, $G_{1}={\left[0,0,-Mg\right]}^{⊤}$ represents the gravitational force acting on the UAV, $G_{2}={\left[0,0,-m_{p}g\right]}^{⊤}$ is the gravitational force on the payload, $a={\left[\ddot{x},\ddot{y},\ddot{z}\right]}^{⊤}$ and $v={\left[\dot{x},\dot{y},\dot{z}\right]}^{⊤}$ are the translational acceleration and velocity of the UAV, respectively, $K_{a}$ is the aerodynamic drag coefficient matrix, and $a_{p}={\left[{\ddot{x}}_{p},{\ddot{y}}_{p},{\ddot{z}}_{p}\right]}^{⊤}$ is the payload translational acceleration.

The tension force $T_{2}$ acting on the payload subsystem can be equivalently treated as the driving force $F_{t}$, i.e., $T_{2}=F_{t}$. This force vector is expressed in the inertial frame as $F_{t}={\left[F_{tx},F_{ty},F_{tz}\right]}^{⊤}$, which satisfies the transformation:

(6) $$F_{t}=R_{b\to a}{\left[0,0,F_{t}\right]}^{⊤}$$

where $R_{b\to a}$ denotes the rotation matrix from the body-fixed frame to the inertial frame, given by:

(7) $$R_{a\to b}=\left[\begin{array}{ccc} C_{\alpha_{x}}C_{\psi_{p}}\; & -S_{\alpha_{y}}S_{\alpha_{x}}C_{\psi_{p}}-C_{\alpha_{y}}S_{\psi_{p}}\; & -C_{\alpha_{y}}S_{\alpha_{x}}C_{\psi_{p}}+S_{\alpha_{y}}S_{\psi_{p}}\\ C_{\alpha_{x}}S_{\psi_{p}}\; & -S_{\alpha_{y}}S_{\alpha_{x}}S_{\psi_{p}}+C_{\alpha_{y}}C_{\psi_{p}}\; & -C_{\alpha_{y}}S_{\alpha_{x}}S_{\psi_{p}}-S_{\alpha_{y}}C_{\psi_{p}}\\ -S_{\alpha_{x}}\; & S_{\alpha_{y}}C_{\alpha_{x}}\; & C_{\alpha_{y}}C_{\alpha_{x}} \end{array}\right]$$

where the shorthand notation $C_{\theta }=\cos\left(\theta \right)$, $S_{\theta }=\sin\left(\theta \right)$ and $T_{\theta }=\tan\left(\theta \right)$ is used for brevity.

The inverse transformation follows as

(8) $$R_{b\to a}=R_{a\to b}^{⊤}$$

Substituting (6) and (8) into (5) yields the compact form:

(9) $$m_{p}a_{p}=\left[\begin{array}{c} -\cos\alpha_{y}\sin\alpha_{x}\cos\psi_{p}+\sin\alpha_{y}\sin\psi_{p}\\ -\cos\alpha_{y}\sin\alpha_{x}\sin\psi_{p}-\sin\alpha_{y}\cos\psi_{p}\\ \cos\alpha_{y}\cos\alpha_{x} \end{array}\right]F_{t}+G_{2}.$$

As illustrated in Figure 1, the components of $F_{t}={\left[F_{tx},F_{ty},F_{tz}\right]}^{⊤}$ are derived geometrically as:

(10) $$\begin{array}{cc} F_{tz} & =F_{t}\cos\alpha_{y}\cos\alpha_{x},\\ F_{tx} & =F_{tz}\tan\alpha_{x},\\ F_{ty} & =\frac{F_{tz}}{\cos\alpha_{x}}\tan\alpha_{y}. \end{array}$$

Here, $F_{t}=∥F_{t}∥$ represents the magnitude of the tether tension force. Under the assumption of a rigid tether, the UAV and payload experience identical acceleration along the cable direction. Applying Newton’s second law to the UAV, we obtain:

(11) $$F_{t}=m_{p}a_{p}-\left[0,\;0,\;1\right]R_{a\to b}G_{2}=m_{p}a_{p}+mg\cos\alpha_{y}\cos\alpha_{x},$$

where $a_{p}$ denotes the magnitude of the payload acceleration along the tether direction.

## 3. Nested Networked Control Design

To achieve tracking and cargo delivery between the UAV and the truck in air-ground collaborative delivery systems, this study proposes a velocity control and swing angle suppression method based on dynamic decoupling. Unlike traditional tracking strategies, the proposed approach, from a networked control perspective, computes the required cable tension from sensor-derived measurements of the desired load velocity and swing angle. Subsequently, motion commands for the UAV body are generated, forming a complete control loop.

### 3.1. Control Network Structure Design

**Figure 2:**
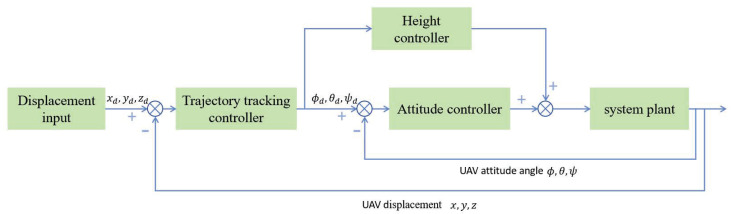
The cascaded control structure of UAV.

As a typical underactuated system, the quadrotor UAV achieves six-degree-of-freedom motion using only the thrust provided by four rotors. As shown in Figure 2, the UAV itself adopts a cascaded control structure, with the outer loop being position control and the input being the desired UAV position $\left(x_{d},y_{d},z_{d}\right)$, and the inner loop being attitude control with the input being the desired UAV attitude angles $\left(\phi_{d},\theta_{d},\psi_{d}\right)$. Although this control structure can achieve basic flight control, it cannot address the challenges posed by suspended payloads.

**Figure 3:**
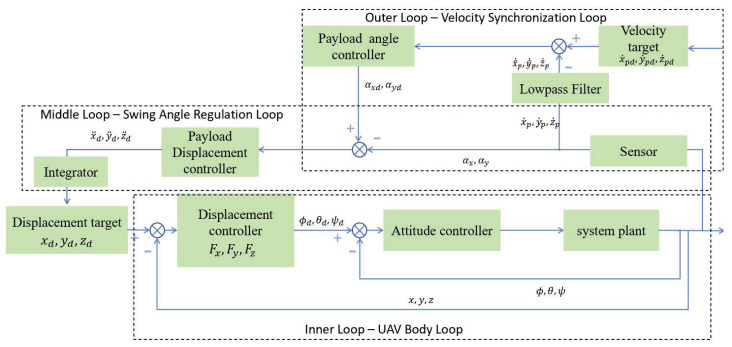
The three-layer nested networked control architecture.

To achieve synchronized constant-speed control of the UAV and the load, this paper constructs a three-layer nested networked control architecture as shown in Figure 3. In this architecture, the load velocity is treated as the primary control objective and placed in the outermost layer, while the UAV position loop serves as the innermost actuator. Since the load has no direct actuation, its motion is indirectly regulated through the swing angle of the sling. Therefore, the swing angle is regarded as a virtual actuator, and a middle transition loop is built around it. The specific design is as follows:

- Outer Loop—Velocity Synchronization Loop
  Taking the load velocity error as input, this loop generates the desired tension force of the sling in real time, and further computes the required swing angle command and its derivative (desired angular velocity).
- Middle Loop—Swing Angle Regulation Loop
  This loop transforms the desired swing angle into a desired angular acceleration. Through a designed virtual angular acceleration controller, it maps the desired angular acceleration into a translational acceleration command required by the UAV, which is then executed by the UAV position controller.
- Inner Loop—UAV Body Loop
  Integrating the UAV position and attitude controllers, this loop accurately tracks the acceleration command provided by the middle loop, ultimately accomplishing the overall velocity synchronization and stable flight mission of the UAV–load system.

### 3.2. Velocity Synchronization Loop Design

As shown in Figure 3, the desired payload velocity is defined as $v_{\mathrm{pd}}={\left[{\dot{x}}_{pd},{\dot{y}}_{pd},{\dot{z}}_{pd}\right]}^{⊤}$, and the desired payload acceleration as $a_{\mathrm{pd}}=\delta ={\left[{\ddot{x}}_{pd},{\ddot{y}}_{pd},{\ddot{z}}_{pd}\right]}^{⊤}$.

In practice, the payload position, swing angles, and other quantities are derived from sensor measurements, which incorporate a significant amount of noise. To mitigate the effects of sensor noise, a first-order low-pass filter [21] is introduced for estimating the velocity. The filtered velocity estimate ${\hat{v}}_{p}$ is obtained from the raw measurements using a first-order filter with a time constant τ>0:

(12) $$\tau {\dot{\hat{v}}}_{p}\left(t\right)+{\hat{v}}_{p}\left(t\right)=v_{p}\left(t\right)$$

or equivalently,

(13) $$\tau \frac{d}{dt}\left({\hat{v}}_{p}\left(t\right)\right)+{\hat{v}}_{p}\left(t\right)=v_{p}\left(t\right)$$

Here, $v_{p}\left(t\right)$ represents the measured velocity of the payload, and ${\hat{v}}_{p}\left(t\right)$ is the estimated velocity after filtering. The estimated payload velocity is given by ${\hat{v}}_{p}={\left[{\dot{\hat{x}}}_{p},{\dot{\hat{y}}}_{p},{\dot{\hat{z}}}_{p}\right]}^{⊤}$, and the desired payload acceleration is ${\hat{a}}_{p}={\left[{\ddot{\hat{x}}}_{p},{\ddot{\hat{y}}}_{p},{\ddot{\hat{z}}}_{p}\right]}^{⊤}$.

This method effectively reduces the velocity estimation errors caused by sensor noise, thereby enhancing the system’s stability and accuracy.

The velocity error variable of the payload is defined as $e_{\mathrm{pv}}$:

(14) $$e_{\mathrm{pv}}=\left[\begin{array}{c} e_{{\dot{x}}_{p}}\\ e_{{\dot{y}}_{p}}\\ e_{{\dot{z}}_{p}} \end{array}\right]=\left[\begin{array}{c} {\dot{x}}_{pd}\\ {\dot{y}}_{pd}\\ {\dot{z}}_{pd} \end{array}\right]-\left[\begin{array}{c} {\dot{\hat{x}}}_{p}\\ {\dot{\hat{y}}}_{p}\\ {\dot{\hat{z}}}_{p} \end{array}\right]$$

The acceleration error variable $e_{\mathrm{pa}}$ is defined as:

(15) $$e_{\mathrm{pa}}=\left[\begin{array}{c} e_{{\ddot{x}}_{p}}\\ e_{{\ddot{y}}_{p}}\\ e_{{\ddot{z}}_{p}} \end{array}\right]=\left[\begin{array}{c} {\ddot{x}}_{pd}\\ {\ddot{y}}_{pd}\\ {\ddot{z}}_{pd} \end{array}\right]-\left[\begin{array}{c} {\ddot{\hat{x}}}_{p}\\ {\ddot{\hat{y}}}_{p}\\ {\ddot{\hat{z}}}_{p} \end{array}\right]$$

The expected value of $F_{t}$ in (11) is defined as $F_{\mathrm{td}}$, and the corresponding desired payload acceleration is designed as the input of the velocity controller along three axes in the payload coordinate system, which can be expressed as:

(16) $$F_{\mathrm{td}}=\left[\begin{array}{c} F_{tx}\\ F_{ty}\\ F_{tz} \end{array}\right]=e_{\mathrm{pa}}+k_{\mathrm{pv}}e_{\mathrm{pv}}+\left[\begin{array}{c} 0\\ 0\\ mg \end{array}\right]=m_{p}\left(e_{\mathrm{pa}}+k_{\mathrm{pv}}e_{\mathrm{pv}}\right)+\left[\begin{array}{c} 0\\ 0\\ mg \end{array}\right]$$

where $k_{\mathrm{pv}}={\left[k_{{\dot{x}}_{p}},k_{{\dot{y}}_{p}},k_{{\dot{z}}_{p}}\right]}^{T}$, and $k_{{\dot{x}}_{p}},k_{{\dot{y}}_{p}},k_{{\dot{z}}_{p}}$ are all non-negative parameters.

The proposed definition enables a systematic design approach: based on the current velocity and acceleration derived from sensor measurements, the desired cable tension is computed and subsequently utilized for the design of the swing angle regulation loop.

### 3.3. Swing Angle Regulation Loop Design

Based on the geometric relationship and Equation (16), the desired swing angles of the payload, $\alpha_{xd}$ and $\alpha_{yd}$, can be inversely solved as follows:

(17) $$\begin{array}{cc} \alpha_{xd} & =\arctan\left(\frac{F_{txd}}{F_{tzd}}\right)\\ \alpha_{yd} & =\arctan\left(\frac{\cos\alpha_{x}F_{tyd}}{F_{tzd}}\right) \end{array}$$

The desired swing angles of the payload, $\alpha_{xd}$ and $\alpha_{yd}$, are obtained through the nested network control design, with their formulation derived from sensor measurements involved in the design process of the previous section. To mitigate sensor noise, the following low-pass filter is introduced:

On this basis, the design of angle velocity and acceleration can be carried out. First, the angle error variable $e_{\alpha }$ is defined as:

(18) $$e_{\alpha }=\left[\begin{array}{c} e_{\alpha_{x}}\\ e_{\alpha_{y}} \end{array}\right]=\left[\begin{array}{c} \alpha_{xd}\\ \alpha_{yd} \end{array}\right]-\left[\begin{array}{c} \alpha_{x}\\ \alpha_{y} \end{array}\right]$$

The angular velocity error variable $e_{\dot{\alpha }}$ and the auxiliary angular velocity error variable ${\dot{e}}_{\sigma }$ are defined as follows:

(19) $$e_{\dot{\alpha }}=\left[\begin{array}{c} e_{{\dot{\alpha }}_{x}}\\ e_{{\dot{\alpha }}_{y}} \end{array}\right]=\left[\begin{array}{c} {\dot{\alpha }}_{xd}\\ {\dot{\alpha }}_{yd} \end{array}\right]-\left[\begin{array}{c} {\dot{\alpha }}_{x}\\ {\dot{\alpha }}_{y} \end{array}\right]$$

The angular velocity controller is designed as:

(20) $${\dot{e}}_{\sigma }=e_{\dot{\alpha }}+k_{\alpha }e_{\alpha }$$

The angular acceleration error variable $e_{\ddot{\alpha }}$ and the auxiliary angular acceleration error variable ${\ddot{e}}_{\sigma }$ are defined as:

(21) $$e_{\ddot{\alpha }}=\left[\begin{array}{c} e_{{\ddot{\alpha }}_{x}}\\ e_{{\ddot{\alpha }}_{y}} \end{array}\right]=\left[\begin{array}{c} {\ddot{\alpha }}_{xd}-{\ddot{\alpha }}_{x}\\ {\ddot{\alpha }}_{yd}-{\ddot{\alpha }}_{y} \end{array}\right]$$

The angular acceleration controller is designed as:

(22) $${\ddot{e}}_{\sigma }=e_{\ddot{\alpha }}+k_{\alpha }\left({\dot{e}}_{\sigma }-k_{\alpha }e_{\alpha }\right)$$

where $k_{\alpha }=\left[\begin{array}{cc} k_{\alpha_{x}} & 0\\ 0 & k_{\alpha_{y}} \end{array}\right]$, $k_{\alpha_{x}}$ and $k_{\alpha_{y}}$ are the proportional gains of the controller.

In this paper, the desired angular acceleration of the payload is set to zero, and no control design is implemented for the torsional acceleration about the cable direction. Consequently, the two-degree-of-freedom form of the angular acceleration ${\ddot{\zeta }}_{p}$ is denoted as ${\ddot{\zeta }}_{d}$:

(23) $${\ddot{\zeta }}_{d}={\left[{\ddot{\phi }}_{p},\;{\ddot{\theta }}_{p}\right]}^{⊤}$$

A virtual payload angular acceleration controller is then defined as:

(24) $${\ddot{\zeta }}_{d}=k_{pa}e_{\alpha }+\left(k_{\alpha }+k_{\dot{\alpha }}\right){\dot{e}}_{\sigma }$$

where $k_{pa},k_{\alpha },k_{\dot{\alpha }}$ also consist of positive controller gains to be determined.

This control law achieves the desired angular velocity and acceleration, and is utilized in the design of the UAV’s inner loop.

### 3.4. UAV Body Loop Design

In order to plan the desired acceleration of the UAV body, ${\ddot{x}}_{d},{\ddot{y}}_{d},{\ddot{z}}_{d}$, it is necessary to analyze the dynamics of the payload. By using the desired velocity of the payload to calculate the desired cable tension, the desired velocity and position targets of the UAV body can be obtained, thereby establishing the relationship with the UAV body state. The dynamic equations of the payload at two different angles $\alpha_{x}$, $\alpha_{y}$ are:

(25) $$\begin{array}{c} L{\ddot{\alpha }}_{x}C_{\alpha_{y}}^{2}=-{\ddot{x}}_{d}C_{\alpha_{x}}-{\ddot{z}}_{d}S_{\alpha_{x}}+2L{\dot{\alpha }}_{x}{\dot{\alpha }}_{y}S_{\alpha_{y}}-gS_{\alpha_{x}},\\ L{\ddot{\alpha }}_{y}={\ddot{x}}_{d}S_{\alpha_{x}}S_{\alpha_{y}}-{\ddot{y}}_{d}C_{\alpha_{y}}-{\ddot{z}}_{d}C_{\alpha_{x}}S_{\alpha_{y}}-L{\dot{\alpha }}_{x}^{2}S_{\alpha_{y}}C_{\alpha_{y}}-gC_{\alpha_{x}}S_{\alpha_{y}}. \end{array}$$

where the shorthand notation $C_{\alpha_{y}}=\cos\left(\alpha_{y}\right)$, $S_{\alpha_{x}}=\sin\left(\alpha_{x}\right)$ is used for brevity.

On the other hand, since the cable between the UAV and the payload is assumed to be rigid, there exists a certain kinematic relationship between the accelerations of the UAV and the payload. The relationship between the payload acceleration along the cable direction and the target acceleration of the UAV, ${\ddot{x}}_{d},{\ddot{y}}_{d},{\ddot{z}}_{d}$, is given by:

(26) $$\begin{array}{cc} a_{p} & =\left[0,\;0,\;1\right]\;R_{b\to a}{\left[{\ddot{x}}_{d},{\ddot{y}}_{d},{\ddot{z}}_{d}\right]}^{T}\\ \; & ={\left[\begin{array}{c} -C_{\alpha_{y}}S_{\alpha_{x}}C_{\psi_{p}}+S_{\alpha_{y}}S_{\psi_{p}}\\ -C_{\alpha_{y}}S_{\alpha_{x}}S_{\psi_{p}}-S_{\alpha_{y}}C_{\psi_{p}}\\ C_{\alpha_{y}}C_{\alpha_{x}} \end{array}\right]}^{\;\;T}{\left[{\ddot{x}}_{d},{\ddot{y}}_{d},{\ddot{z}}_{d}\right]}^{T} \end{array}$$

Through a process of dynamic inversion and geometric analysis that combines the relationships in Equations (7), (8), (25) and (26), we arrive at the following control law for the desired UAV acceleration:

(27) $$\begin{array}{cc} {\ddot{z}}_{d} & =C_{\alpha_{x}}C_{\alpha_{y}}[a_{p}+\left(2L{\dot{\alpha }}_{x}{\dot{\alpha }}_{y}S_{\alpha_{y}}S_{\alpha_{y}}-gS_{\alpha_{x}}S_{\alpha_{y}}\right)\\ \; & \;-{\left(\sec\alpha_{y}\right)}^{2}\left(L{\dot{\alpha }}_{x}^{2}S_{\alpha_{y}}C_{\alpha_{y}}+gC_{\alpha_{x}}S_{\alpha_{y}}\right)T_{\alpha_{x}}-\left(L-\frac{1}{m_{p}L}\right){\ddot{\alpha }}_{x}T_{\alpha_{x}}],\\ {\ddot{x}}_{d} & ={\ddot{z}}_{d}T_{\alpha_{x}}+\frac{LC_{\alpha_{y}}C_{\alpha_{y}}-L{\dot{\alpha }}_{x}^{2}S_{\alpha_{y}}C_{\alpha_{y}}-gC_{\alpha_{x}}S_{\alpha_{y}}}{C_{\alpha_{x}}C_{\alpha_{y}}},\\ {\ddot{y}}_{d} & =\left(2L{\dot{\alpha }}_{x}{\dot{\alpha }}_{y}S_{\alpha_{y}}C_{\alpha_{y}}-gS_{\alpha_{x}}C_{\alpha_{y}}\right)C_{\alpha_{y}}-a_{p}S_{\alpha_{y}}-L{\ddot{\alpha }}_{y}C_{\alpha_{y}}. \end{array}$$

The above expressions include the analytical relationship between the UAV and payload dynamics. If precise control of the payload velocity is required, it is necessary to design not only for $a_{p}$, but also for the swing angular accelerations ${\ddot{\alpha }}_{x}$ and ${\ddot{\alpha }}_{y}$.

For the design of $a_{p}$, the expression in Equation (11) can be further derived. Based on the angular acceleration error defined in Equation (21), the virtual control input ${\ddot{e}}_{\sigma }$ needs to be mapped to the motion command of the UAV body. According to the dynamic analytical relationship in Equation (27), the desired acceleration of the UAV can be expressed as:

(28) $$a_{d}=f\left(e_{pv},{\dot{e}}_{\sigma },{\ddot{e}}_{\sigma },F_{td}\right)$$

where the function f(·) is analytically determined by Equation (27). It shows that the target acceleration of the UAV position is jointly determined by the payload velocity, swing angle velocity, swing angle acceleration, and cable tension.

After substituting the virtual control input from Equation (25), the desired UAV acceleration $a_{d}$ can be decomposed into three components $\left({\ddot{x}}_{d},{\ddot{y}}_{d},{\ddot{z}}_{d}\right)$. Through integration, the desired UAV trajectory $\xi_{d}={\left[x_{d},y_{d},z_{d}\right]}^{⊤}$ and ${\dot{\xi }}_{d}={\left[{\dot{x}}_{d},{\dot{y}}_{d},{\dot{z}}_{d}\right]}^{⊤}$ can be obtained.

**Figure 4:**
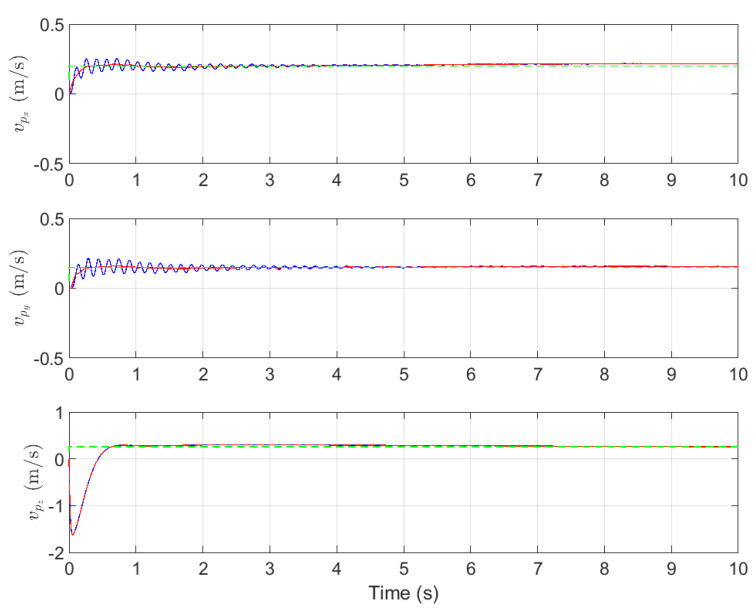
The velocity response.

In the inner loop of the nested control architecture shown in Figure 4, the UAV position tracking error is defined as $e_{\xi }=\xi_{d}-\xi$ and ${\dot{e}}_{\xi }={\dot{\xi }}_{d}-\dot{\xi }$. The UAV position controller adopts PD control with the following control forces:

(29) $$\begin{array}{cc} F_{x} & =k_{px_{0}}e_{\xi x}+k_{dx_{0}}{\dot{e}}_{\xi x}\\ F_{y} & =k_{py_{0}}e_{\xi y}+k_{dy_{0}}{\dot{e}}_{\xi y}\\ F_{z} & =k_{pz_{0}}e_{\xi z}+k_{dz_{0}}{\dot{e}}_{\xi z} \end{array}$$

where $e_{\xi }={\left[e_{\xi x},e_{\xi y},e_{\xi z}\right]}^{⊤}$, ${\dot{e}}_{\xi }={\left[{\dot{e}}_{\xi x},{\dot{e}}_{\xi y},{\dot{e}}_{\xi z}\right]}^{⊤}$, and $k_{px_{0}},k_{dx_{0}},k_{py_{0}},k_{dy_{0}},k_{pz_{0}},k_{dz_{0}}$ are positive definite controller gains.

This control law ensures accurate tracking of the desired trajectory generated by the outer loops, thereby enabling simultaneous velocity synchronization and payload swing suppression through the nested control structure.

## 4. Stability Analysis

The complete error state vector E that encompasses all tracking errors in the nested control architecture is defined as:

(30) $$E=\left[\begin{array}{c} e_{\xi }\\ {\dot{e}}_{\xi }\\ e_{pv}\\ e_{\alpha }\\ {\dot{e}}_{\sigma }\\ e_{\delta } \end{array}\right]=\left[\begin{array}{c} \xi_{d}-\xi \\ {\dot{\xi }}_{d}-\dot{\xi }\\ v_{pd}-v_{p}\\ \alpha_{d}-\alpha \\ {\dot{\alpha }}_{d}-\dot{\alpha }+K_{\alpha }\left(\alpha_{d}-\alpha \right)\\ \delta_{d}-\delta \end{array}\right]$$

where $e_{\xi },{\dot{e}}_{\xi }$ represent UAV position and velocity tracking errors, $e_{pv}$ is the payload velocity tracking error defined in (14), $e_{\alpha }$ denotes the payload swing angle tracking error defined in (18), ${\dot{e}}_{\sigma }$ corresponds to the augmented angular velocity error from the virtual controller defined in (20), and $e_{\delta }$ represents the payload position tracking error.

The desired states in this nested control hierarchy are generated through explicit mathematical relationships derived from the control design:

(31) $$\begin{array}{cc} \alpha_{d} & =\left[\begin{array}{c} \arctan\left(\frac{F_{txd}}{F_{tzd}}\right)\\ \arctan\left(\frac{\cos\alpha_{x}F_{tyd}}{F_{tzd}}\right) \end{array}\right]\;(\mathrm{from}\;(17))\\ {\dot{\alpha }}_{d} & =\dot{\alpha }+e_{\dot{\alpha }}+K_{\alpha }e_{\alpha }\;(\mathrm{from}\;(20)\;\mathrm{and}\;(19))\\ \xi_{d} & =\int \int a_{d}dt^{2}\;\mathrm{where}\;a_{d}=f\left(e_{pv},{\dot{e}}_{\sigma },{\ddot{e}}_{\sigma },F_{td}\right)\;\mathrm{from}\;(28)\\ {\dot{\xi }}_{d} & =\int a_{d}dt=\int f\left(e_{pv},{\dot{e}}_{\sigma },{\ddot{e}}_{\sigma },F_{td}\right)dt\;(\mathrm{from}\;(28)) \end{array}$$

These mappings represent the hierarchical information flow in the nested control architecture, demonstrating how the outer loop objectives (velocity synchronization) are transformed through successive layers into inner loop commands (UAV position and velocity references).

To establish the closed-loop stability, we first derive the error dynamics for each subsystem. Starting with the UAV error dynamics obtained by combining (4) with the control law (29):

(32) $$\begin{array}{cc} M{\ddot{e}}_{\xi } & =M{\ddot{\xi }}_{d}-F+T_{1}+K_{a}\dot{\xi }-G_{1}\\ \; & =M{\ddot{\xi }}_{d}-\left[K_{p}e_{\xi }+K_{d}{\dot{e}}_{\xi }\right]+T_{1}+K_{a}\left({\dot{\xi }}_{d}-{\dot{e}}_{\xi }\right)-G_{1} \end{array}$$

The payload error dynamics follow directly from (5):

(33) $$m_{p}{\ddot{e}}_{\delta }=m_{p}{\ddot{\delta }}_{d}-T_{2}-G_{2}$$

For the swing angle dynamics, we express (25) in error coordinates:

(34) $$\begin{array}{cc} L\left({\ddot{\alpha }}_{xd}-{\ddot{e}}_{\alpha x}\right)C_{\alpha_{y}}^{2}= & -\left({\ddot{x}}_{d}-{\ddot{e}}_{\xi x}\right)C_{\alpha_{x}}-\left({\ddot{z}}_{d}-{\ddot{e}}_{\xi z}\right)S_{\alpha_{x}}\\ \; & +2L\left({\dot{\alpha }}_{xd}-{\dot{e}}_{\alpha x}\right)\left({\dot{\alpha }}_{yd}-{\dot{e}}_{\alpha y}\right)S_{\alpha_{y}}-gS_{\alpha_{x}} \end{array}$$

The velocity error dynamics are derived from the definition and the tension relationship in (11):

(35) $${\dot{e}}_{pv}={\dot{v}}_{pd}-\frac{1}{m_{p}}\left(T_{2}+G_{2}\right)$$

Based on this interconnection structure, the complete error dynamics can be compactly represented as:

(36) $$\dot{E}=A\left(E\right)E+B\left(E\right)u+\Delta \left(E\right)$$

where A(E) denotes the state-dependent system matrix capturing the nonlinear dynamics, B(E) represents the input matrix mapping control actions, $u={\left[F^{⊤},F_{td}^{⊤}\right]}^{⊤}$ combines control inputs from (29) and (16), and Δ(E) encompasses bounded disturbance terms including sensor noise and external disturbances.

To analyze the system stability, we construct a composite Lyapunov function that accounts for all energy and tracking errors:

(37) $$\begin{array}{cc} V(E)= & \frac{1}{2}{\dot{e}}_{\xi }^{⊤}M{\dot{e}}_{\xi }+\frac{1}{2}e_{\xi }^{⊤}K_{p}e_{\xi }+e_{\xi }^{⊤}K_{d}{\dot{e}}_{\xi }\\ \; & +\frac{1}{2}e_{pv}^{⊤}P_{v}e_{pv}+\frac{1}{2}e_{\alpha }^{⊤}P_{\alpha }e_{\alpha }+\frac{1}{2}{\dot{e}}_{\sigma }^{⊤}P_{\sigma }{\dot{e}}_{\sigma }\\ \; & +\frac{1}{2}m_{p}{\dot{e}}_{\delta }^{⊤}{\dot{e}}_{\delta }+\frac{1}{2}e_{\delta }^{⊤}K_{\delta }e_{\delta }+V_{\mathrm{coupling}}\left(E\right) \end{array}$$

where the coupling energy term $V_{\mathrm{coupling}}\left(E\right)=\frac{1}{2}k_{t}{∥\xi -\delta -Le_{r}∥}^{2}$ captures the energy in the cable coupling, with $e_{r}$ being the unit vector along the cable and $k_{t}>0$ a virtual spring constant representing the cable tension. This term explicitly accounts for the geometric constraint imposed by the rigid cable assumption.

Differentiating V(E) along the trajectories of the complete error system yields:

(38) $$\begin{array}{cc} \dot{V}\left(E\right)= & {\dot{e}}_{\xi }^{⊤}M{\ddot{e}}_{\xi }+{\dot{e}}_{\xi }^{⊤}K_{p}e_{\xi }+{\ddot{e}}_{\xi }^{⊤}K_{d}{\dot{e}}_{\xi }+{\dot{e}}_{\xi }^{⊤}K_{d}{\ddot{e}}_{\xi }\\ \; & +{\dot{e}}_{pv}^{⊤}P_{v}e_{pv}+{\dot{e}}_{\alpha }^{⊤}P_{\alpha }e_{\alpha }+{\ddot{e}}_{\sigma }^{⊤}P_{\sigma }{\dot{e}}_{\sigma }+{\dot{e}}_{\sigma }^{⊤}P_{\sigma }{\ddot{e}}_{\sigma }\\ \; & +m_{p}{\dot{e}}_{\delta }^{⊤}{\ddot{e}}_{\delta }+{\dot{e}}_{\delta }^{⊤}K_{\delta }e_{\delta }+{\dot{V}}_{\mathrm{coupling}} \end{array}$$

Substituting the error dynamics from (32)–(35), and after extensive algebraic manipulation that accounts for the specific form of the coupling energy derivative, we obtain the bound:

(39) $$\dot{V}\left(E\right)\leq -E^{⊤}\Gamma \left(E\right)E+E^{⊤}\Delta \left(E\right)$$

where Γ(E)>0 is a state-dependent positive definite matrix when the control gains satisfy:

(40) $$\begin{array}{cc} \lambda_{\min}\left(K_{p}\right) & >\gamma_{1},\;\lambda_{\min}\left(K_{d}\right)>\gamma_{2}\\ \lambda_{\min}\left(K_{pv}\right) & >\gamma_{3},\;\lambda_{\min}\left(K_{\alpha }\right)>\gamma_{4}\\ \lambda_{\min}\left(K_{pa}\right) & >\gamma_{5},\;\lambda_{\min}\left(K_{a}+K_{d}\right)>\gamma_{6} \end{array}$$

for sufficiently large constants $\gamma_{1},\ldots ,\gamma_{6}>0$ that depend on the system parameters and the maximum expected disturbances.

The disturbance term Δ(E) incorporates the effects of sensor noise characterized in (12) and sudden external disturbances considered in the simulation studies. Since Δ(E) is bounded and vanishes at E=0, and Γ(E) remains positive definite under the gain conditions (40), we conclude by Lyapunov’s direct method that the equilibrium E=0 is uniformly asymptotically stable.

This stability result demonstrates that the proposed three-layer nested control architecture guarantees convergence of all tracking errors to zero while maintaining robustness against sensor noise and external disturbances, thereby achieving the dual control objectives of velocity synchronization and swing angle suppression.

## 5. Simulation Experiments and Results Analysis

The effectiveness of the proposed three-layer nested networked control architecture is validated through numerical simulations, which account for both random sensor noise of 1/1000 amplitude and sudden external disturbances introduced at t = 5 s. First, the drone–payload system is tasked with tracking a ground vehicle that follows a step-change in velocity reference. The transient response, swing angle convergence rate, and residual oscillation amplitude are analyzed. Subsequently, the system’s robustness is evaluated under external disturbances. In both simulation scenarios, the following performance metrics are assessed: the convergence speed and magnitude of the swing angle, the time required to achieve the desired payload velocity, and the overall tracking accuracy.

In the simulations, the drone mass *M*, payload mass $m_{p}$, rotor-to-rotor distance *r*, and cable length *L* are set to M=0.8kg, $m_{p}=0.06\;\mathrm{kg}$, r=0.3m, L=0.6m. The UAV and the load are both initially at rest: ${\dot{x}}_{0}=0\;m/s$, ${\dot{y}}_{0}=0\;m/s$, ${\dot{z}}_{0}=0\;m/s$, ${\dot{x}}_{p0}=0\;m/s$, ${\dot{y}}_{p0}=0\;m/s$, ${\dot{z}}_{p0}=0\;m/s$. The desired payload-velocity reference is set to ${\dot{x}}_{pd}=0.2\;m/s,$${\dot{y}}_{pd}=0.2\;m/s,{\dot{z}}_{pd}=0.2\;m/s$. The load-velocity controller gains are $k_{\dot{x}p}=0.01$, $k_{\dot{y}p}=0.01$, $k_{\dot{z}p}=0.01$. The load-side virtual controller parameters are $k_{\alpha_{x}}=1$, $k_{\alpha_{y}}=1$, $k_{\dot{\alpha }x}=1$, $k_{\dot{\alpha }y}=2$. The UAV position controller parameters are $k_{px}=5$, $k_{dx}=6$, $k_{py}=12$, $k_{dy}=22$, $k_{pz}=2$, $k_{dz}=4$.

**Figure 5:**
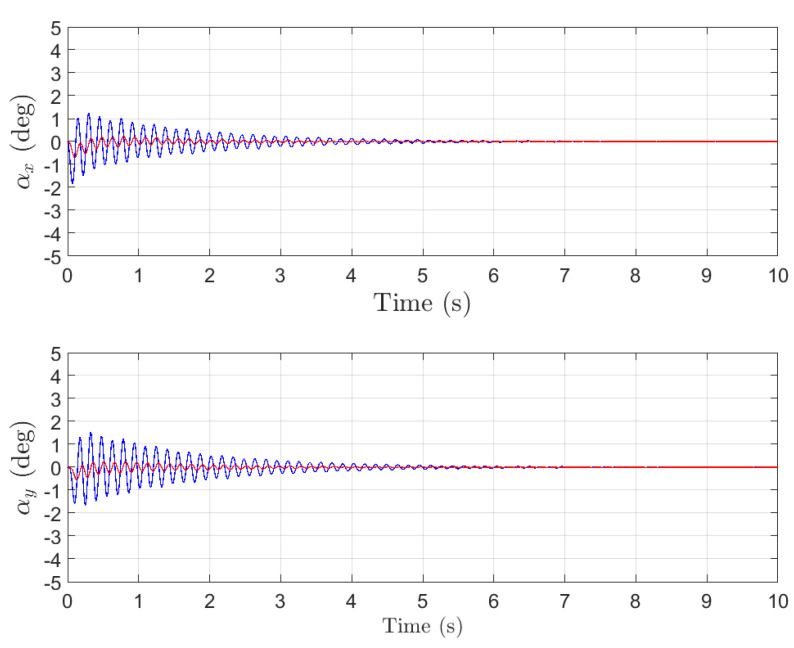
The angles response.

With the coupled system model given in (4) and (9), and the control algorithm proposed in this paper, numerical simulations are carried out. The system responses are shown in Figure 4 and Figure 5. In Figure 4, the three subplots illustrate the velocity responses of the payload in three dimensions. The blue curve represents the velocity response of the load $\left({\dot{x}}_{p},{\dot{y}}_{p},{\dot{z}}_{p}\right)$ from sensor measurement, the red curve represents the velocity response of the UAV $\left({\dot{x}}_{o},{\dot{y}}_{o},{\dot{z}}_{o}\right)$ with low pass filter, and the green curve represents the reference velocity target $\left({\dot{x}}_{pd},{\dot{y}}_{pd},{\dot{z}}_{pd}\right)$, which corresponds to the motion of a ground vehicle acting as the target for the load. In Figure 5, the blue curve represents the variation of the two swing angles of the load, $\alpha_{x}$ and $\alpha_{y}$, from sensor measurement, while the red curve represents the actually swing angle response with the filtered signals.

The simulation results show that the proposed control scheme achieves both velocity tracking and swing angle suppression performance. As shown in Figure 4, the load velocity can accurately track the UAV motion, where the horizontal direction (*x*) and vertical direction (*y*) all reach the target velocity within 1 s. Meanwhile, as seen from Figure 5, within 2 s after the system is activated, the load’s swing angle quickly converges to within 0.2° (with the maximum instantaneous swing less than 2°). Simulation results demonstrate that the control system simultaneously achieves rapid velocity tracking and effective swing suppression, thereby fulfilling the dual control objectives.

**Figure 6:**
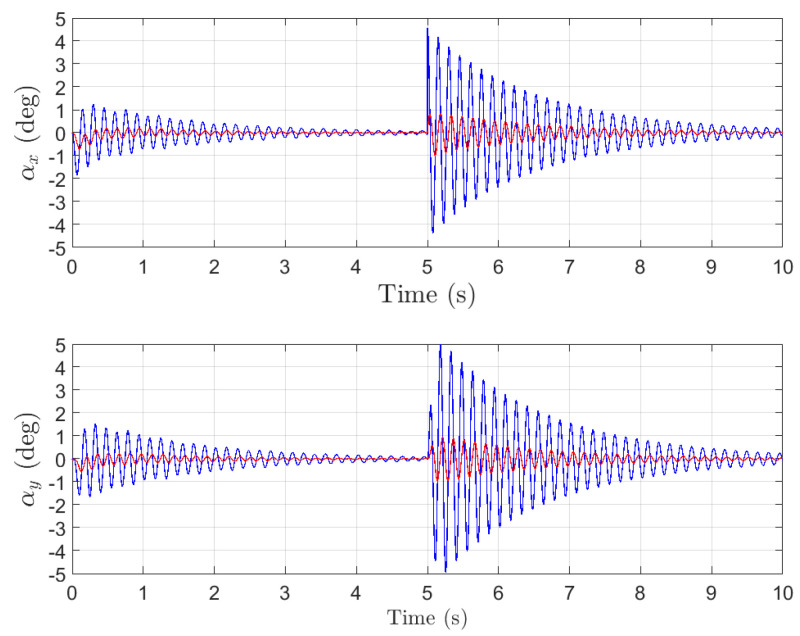
The payload angle response with disturbance.

To further evaluate the robustness of the proposed control strategy, external disturbances are introduced into the simulation. A disturbance is applied to the payload at t=5s, after the system has reached a steady state. The resulting swing angle responses are depicted in Figure 6. As shown in the figure, the proposed controller (red curve) effectively suppresses the induced oscillations, with the swing angles returning to a steady state within 2 s. In contrast, the raw sensor signals (blue curve) exhibit prolonged oscillations, demonstrating that the proposed method significantly enhances the system’s ability to reject disturbances and recover stability.

In closed-loop networks composed of sensors, communication links and controllers, noise and disturbance are not solely exogenous. The control network itself acts as a sophisticated signal conduit that injects computational noise and quantization errors while amplifying and propagating sensor noise throughout the loop. Put differently, the network is an intrinsic source of both interference and sensor noise. The blue traces in Figure 4, Figure 5 and Figure 6 depict the target signal after it has traversed this noisy channel, whereas the red traces show the same signal after the proposed design attenuates these disturbances at the implementation level.

## 6. Conclusions and Future Research Directions

This study addresses the challenge of high-precision cooperative control in air–ground collaborative distribution systems. To enable accurate tracking of moving ground targets while simultaneously suppressing the payload swing, a three-layer nested networked control architecture is proposed. The proposed design explicitly accounts for the underactuated and strongly coupled dynamics of the UAV-slung-load system while incorporating robustness against sensor noise and sudden disturbances. Within this framework, the system is dynamically decomposed into a UAV-driven subsystem and a load swing subsystem. By employing an independent variable separation approach, UAV velocity commands are derived and converted into position references for attitude control. Furthermore, a virtual swing angle controller and an indirect cable tension controller are designed, establishing nonlinear feedback to stabilize the velocity transformation process.

Simulation results demonstrate that the proposed control scheme achieves a UAV-load velocity synchronization error of less than 0.1 m/s while ensuring the payload swing angle converges within 0.2° in 2 s. The integrated strategy of dynamic decoupling and virtual control offers a novel technical pathway for precise velocity regulation in cable-driven UAVs under complex operating conditions. This study demonstrates that the proposed nested control architecture provides an effective solution for enhancing the performance of sensor-enabled networked control systems. It successfully addresses the intertwined challenges of sensor noise mitigation and closed-loop stability maintenance in air–ground collaborative distribution applications.

In the future, further research could be conducted in these directions:

1. **Prescribed-time convergent control.**
  The present nested architecture only guarantees asymptotic convergence. We will incorporate a time-based generator or homogeneous finite-time approach to reshape the outer-loop velocity synchronization layer into a prescribed-time controller, thereby providing an explicit upper bound on the settling time for both swing suppression and velocity tracking. The results will be compared with the fixed-time stabilization of linear control systems [22] and the prescribed finite-time consensus tracking for multiagent systems with nonholonomic chained-form dynamics [23].
2. **Elastic cable and mass uncertainty.**
  The rigid cable assumption will be relaxed to a distributed-mass or visco-elastic model, and adaptive/robust strategies will be developed to handle longitudinal vibrations and coupled UAV–payload dynamics.
3. **Experimental validation and hardware-in-the-loop.**
  A self-developed quadrotor–UGV heterogeneous platform will be used for hardware-in-the-loop tests to quantify the effects of sensor noise and communication packet loss on prescribed-time accuracy. The dataset and code will be open-sourced.

## Data Availability

The dynamic model and the simulation result can be found at https://blog.csdn.net/weixin_56678845/article/details/154032817?spm=1001.2014.3001.5502 (accessed on 28 October 2025).
